# Revision of the Australian guidelines to reduce health risks from drinking alcohol

**DOI:** 10.5694/mja2.51336

**Published:** 2021-11-28

**Authors:** Katherine M Conigrave, Robert L Ali, Rebecca Armstrong, Tanya N Chikritzhs, Peter d’Abbs, Mark F Harris, Nicole Hewlett, Michael Livingston, Dan I Lubman, Anne McKenzie, Colleen O’Leary, Alison Ritter, Scott Wilson, Melanie Grimmond, Emily Banks

**Affiliations:** ^1^ Royal Prince Alfred Hospital Sydney NSW; ^2^ University of Sydney Sydney NSW; ^3^ University of Adelaide Adelaide SA; ^4^ University of Melbourne Melbourne VIC; ^5^ National Drug Research Institute Curtin University Perth WA; ^6^ Menzies School of Health Research Darwin NT; ^7^ Centre for Primary Health Care and Equity University of New South Wales Sydney NSW; ^8^ Centre for Healthcare Transformation Queensland University of Technology Brisbane QLD; ^9^ Turning Point Eastern Health Melbourne VIC; ^10^ Monash Addiction Research Centre Monash University Melbourne VIC; ^11^ Telethon Kids Institute Perth WA; ^12^ Office of the Chief Psychiatrist of Western Australia Perth WA; ^13^ Drug Policy Modelling Program Social Policy Research Centre UNSW Sydney Sydney NSW; ^14^ Aboriginal Drug and Alcohol Council SA Aboriginal Corporation Adelaide SA; ^15^ National Health and Medical Research Council Canberra ACT; ^16^ National Centre for Epidemiology and Population Health Australian National University Canberra ACT

**Keywords:** Policy, drugs and alcohol, Alcohol‐related disorders, Prevention and control, Health communication, Preventive medicine

## Abstract

**Introduction:**

The *Australian guidelines to reduce health risks from drinking alcohol* were released in 2020 by the National Health and Medical Research Council. Based on the latest evidence, the guidelines provide advice on how to keep the risk of harm from alcohol low. They refer to an Australian standard drink (10 g ethanol).

**Recommendations:**

• Guideline 1: To reduce the risk of harm from alcohol‐related disease or injury, healthy men and women should drink no more than ten standard drinks a week and no more than four standard drinks on any one day. The less you drink, the lower your risk of harm from alcohol.
• Guideline 2: To reduce the risk of injury and other harms to health, children and people under 18 years of age should not drink alcohol.
• Guideline 3: To prevent harm from alcohol to their unborn child, women who are pregnant or planning a pregnancy should not drink alcohol. For women who are breastfeeding, not drinking alcohol is safest for their baby.

**Changes as result of the guideline:**

The recommended limit for healthy adults changed from two standard drinks per day (effectively 14 per week) to ten per week. The new guideline states that the less you drink, the lower your risk of harm from alcohol. The recommended maximum on any one day remains four drinks (clarified from previously “per drinking occasion”). Guidance is clearer for pregnancy and breastfeeding, and for people aged less than 18 years, recommending not drinking.

The National Health and Medical Research Council (NHMRC) of Australia is responsible for developing guidelines on a wide range of public, clinical and environmental health issues. Since 1987 this has included guidelines on reducing the health risks of alcohol, with the latest revision released in 2020.^1^

## Drinking and harm in Australia

Alcohol contributes 4.5% of the burden of disease and injury in Australia and is the lead contributor at age 15–44 years.^2^ The pattern of drinking influences harms. For example, consuming 14 standard drinks on a single day increases the risk of acute harms, such as injury and heart rhythm problems, far more than drinking two drinks daily over a week. Both patterns are likely to increase risks of chronic conditions, such as cancer, compared with not drinking. In Australia, the commonest frequency of consumption is 3 days per week.^1^

## Rationale for revising the guidelines on alcohol consumption

Evidence on alcohol and health has evolved considerably since the last update of the NHMRC guidelines in 2009.^3^ Evidence linking alcohol to cancer risk has strengthened, particularly at lower levels of consumption. Alcohol is classed as a Group 1 carcinogen by the International Agency for Research on Cancer, with strong evidence of increased risk of seven types of cancer: breast, colon and rectum, pancreas, liver, oesophagus, mouth, and throat and pharynx.^4^ Most of these cancers demonstrate increasing risk on a continuum of growing alcohol consumption. For example, the risk of breast cancer in women increases by 12% per additional standard drink (10 g ethanol) per day, with elevated risks apparent from an average of one standard drink per day.^5^

Consistent with this changing evidence, internationally recommended limits to reduce risk from alcohol consumption have been decreasing steadily, including recent downward revisions in the United Kingdom, the Netherlands and France.^6^

There is also increasing uncertainty over the extent and veracity of reported protective effects of alcohol against a range of health conditions, including myocardial infarction. Rigorous analysis, limited to cohorts with well defined comparison groups, shows far less, if any, protective effect.^7^ Similarly, Mendelian randomisation studies have also cast doubt over purported protective effects.^8^

## Creating guidelines for a risk that is voluntarily taken

Tight guidelines are typically placed on environmental (involuntary) toxins to keep the risk of dying from that cause at no more than around one in 1 million.^9^ However, individuals typically choose to drink alcohol, despite some awareness of the risk. The 2009 guidelines to reduce health risks from drinking alcohol for the general adult population used drinking thresholds that would limit lifetime (absolute) risk of dying from an alcohol‐related cause to less than one in 100 — similar to the lifetime level of risk of driving a car (data from the United States).^10^ This is very different from choosing the guideline that minimises the risk of alcohol‐related harm, which would give a far lower limit. A guideline that eliminates risks from alcohol altogether would advise no consumption.

In this article, we describe the process of revising the *Australian guidelines to reduce health risks from drinking alcohol* and the resulting guidelines.

## Methods

### Overview of the guidelines revision process

The revision of the guidelines followed standard NHMRC processes, which are similar to the Appraisal of Guidelines for Research and Evaluation (AGREE) II criteria.^11^, ^12^ Key stages of the revision process were:

• Establishing an independent expert advisory committee, comprising 14 members with expertise in epidemiology, sociology, medicine, mental health, addiction, public health, and community engagement.
• Procuring systematic reviews and mathematical modelling to provide evidence on short and long term harms and benefits associated with various levels and patterns of drinking; the population, exposure, comparator and outcome (PECO) criteria and protocols were determined in advance by the committee.
• Applying the Grading of Recommendations, Assessment, Development and Evaluation (GRADE) process to rate quality and certainty of the evidence; the GRADE Evidence to Decision frameworks were used to translate evidence into recommendations and provide transparency around decision making.^13^
• Undertaking public consultation to understand issues of concern for the public and stakeholders and to ensure accountability of the agency, independence of the guidelines, and an improved final guideline.
• Obtaining independent expert reviews of the protocols, systematic reviews, guidelines and mathematical modelling; this helped ensure the guidelines followed the rigorous approach specified in the research protocols, assessed certainty of the evidence on which the recommendations were based and usability and acceptability of recommendations.^14^
• Endorsement of the guidelines by the Council of the NHMRC, whose members include state, territory and Commonwealth chief health and medical officers.

### The evidence base for the guidelines

Multiple sources of evidence were considered by the committee (Box 1). This included:

Box 1 **Schema of inputs used to develop the draft guidelines**

**Figure:**
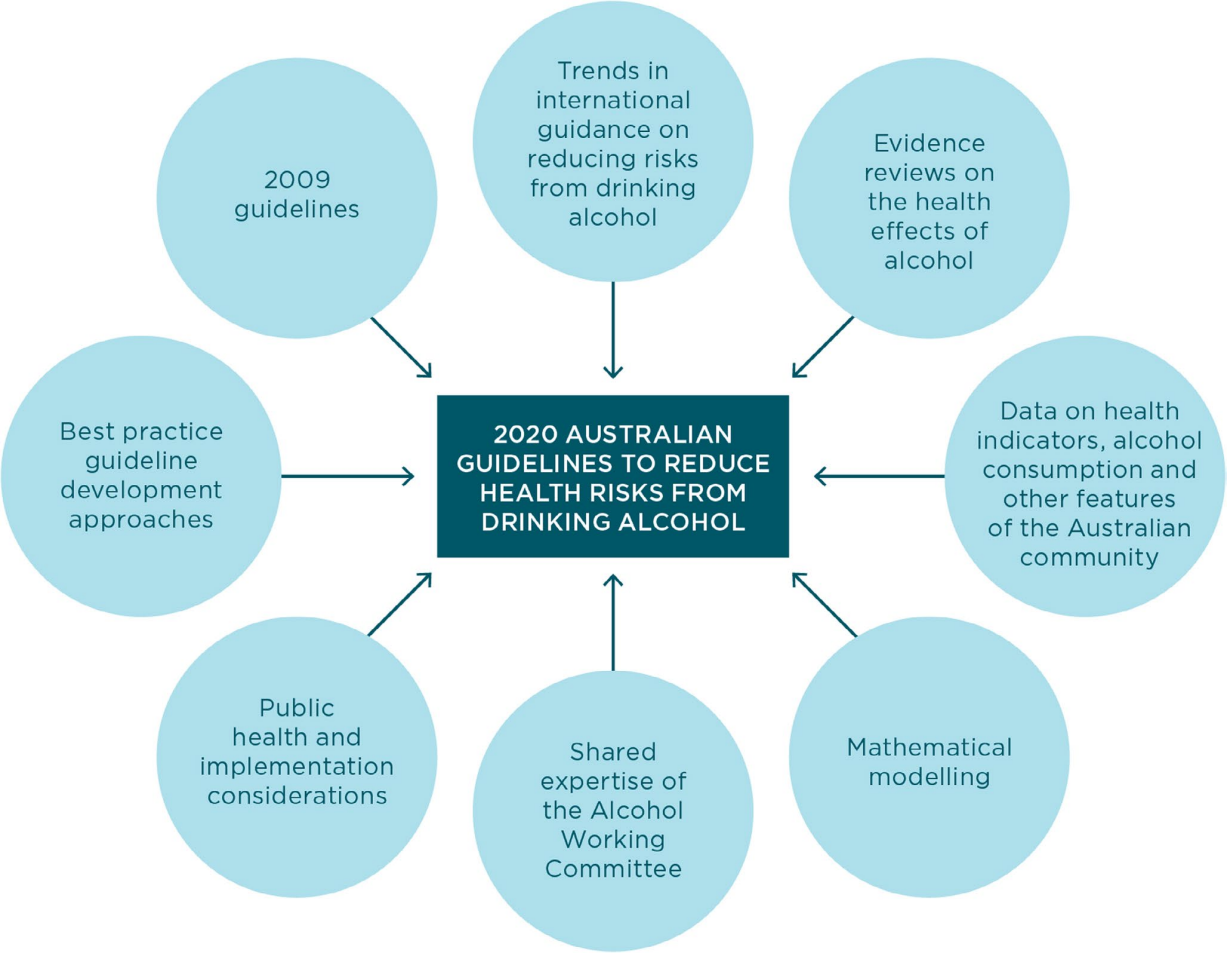
Source: National Health and Medical Research Council.^1^

• the 2009 guidelines;
• international guidance on reducing risks from drinking alcohol;
• evidence reviews on the health effects of alcohol;
• data on health indicators, alcohol consumption and other features of the Australian community; and
• mathematical modelling.

The process also drew upon the expertise of the NHMRC Alcohol Working Committee, and took into account public health and implementation considerations and best practice guideline development approaches.

### The 2009 guidelines

The committee started with the principle that the 2009 guidelines should only be changed if there was compelling evidence of need to do so.

For the guideline for the general adult population, the committee retained the aim to ascertain the upper level of drinking that would limit lifetime (absolute) risk of dying from an alcohol‐related cause to less than one in 100.

### International guidelines

There is considerable variation in guidelines internationally and, as described above, the recommended drinking limits have been reducing. France and the United Kingdom currently recommend drinking no more than the equivalent of ten to 11 Australian standard drinks over a week respectively, while the Netherlands recommends zero or not more than seven per week.^6^

#### The evidence review

An overview of systematic reviews was conducted to consider the short and long term health risks and benefits associated with various levels and patterns of drinking alcohol. This focused on identifying systematic reviews and meta‐analyses published since 2007 to identify areas where evidence has grown since the 2009 guidelines.

Research protocols were first reviewed by the Committee. These outlined the scope; scientific question, including PECO; the search strategy; and methods. Over 50 health outcomes were selected by the Committee as highly relevant for determining the guidelines. Importance to the public of a health outcome was considered in the selection (Box 2).

Box 2 **Health outcomes for which evidence was identified in the overview of systematic reviews and supplementary systematic reviews for the revised *Australian guidelines to reduce health risks from drinking alcohol***

**Table:** 

Health conditions		
**Long term health conditions**		
• All‐cause mortality • Bladder cancer • Brain cancer • Breast cancer (premenopausal) • Breast cancer (postmenopausal) • Cervical cancer • Colorectal cancer • Endometrial cancer • Gallbladder cancer • Kidney cancer • Liver cancer • Lung cancer • Hodgkin lymphoma • Non‐Hodgkin lymphoma	• Leukaemia • Multiple myeloma • Melanoma • Mouth and pharynx cancer • Larynx cancer • Oesophageal squamous cell carcinoma • Oesophageal adenocarcinoma • Ovarian cancer • Pancreatic cancer • Prostate cancer • Stomach cancer • Thyroid cancer • Coronary heart disease • Atrial fibrillation	• Heart failure • Ischaemic stroke • Intracerebral haemorrhage • Subarachnoid haemorrhage • Hypertension • Liver cirrhosis • Pancreatitis • Type 2 diabetes • Dementia and cognitive decline • Seizures • Hip fracture • Gout • Pneumonia • Tuberculosis
**Short term health conditions**		
• Injury • Fatal motor vehicle injury	• Myocardial infarction or coronary event • Ischaemic stroke	• Haemorrhagic stroke
**Mental health conditions**		
• Depression/depressive symptoms • Anxiety/anxiety symptoms	• Bipolar disorder • Suicide/suicide ideation/suicide attempts	• PTSD/PTSD symptoms
**Cognitive impairment conditions**		
• Cognition		
**Pregnancy outcomes**		
• Preterm birth • Small for gestational age • Communication delay	• Behavioural problems • Low birthweight • Child motor function	• Birth defects
**Breastfeeding outcomes**		
• Sedation		

PTSD = post‐traumatic stress disorder.

The systematic reviews focused on the physical and psychological health harms of alcohol use to the individual drinking and to the developing fetuses and babies of mothers who consume alcohol while pregnant or breastfeeding. The reviews did not include social or economic outcomes, despite the Committee acknowledging these harms can be substantial. It was noted that if an individual’s consumption is kept to the recommended guidelines, this would also be likely to reduce the risk of social and economic harms from alcohol.

The overviews of systematic reviews were commissioned from external researchers. Additional systematic reviews were also commissioned if no quality systematic reviews were located, especially if the topic was of high importance. In total, 38 high quality systematic reviews were selected and four additional systematic reviews were commissioned on mental health, long term cognitive impairment, pregnancy and breastfeeding.

The quality and certainty of the evidence was evaluated using the GRADE approach, with modifications for public health studies as described elsewhere.^15^ Other tools were also used to assess the systematic reviews: A MeaSurement Tool to Assess Systematic Reviews (AMSTAR)^16^ and the Risk of Bias in Systematic Reviews (ROBIS).^17^

#### The nature and quality of the evidence

The quality of the evidence included in the systematic reviews varied across outcomes. GRADE rates the type of epidemiological evidence typical of broad public health exposures as low to very low; accordingly, the certainty in the overall evidence was rated as very low. More highly rated study designs that are considered necessary for clinical practice guidelines, such as randomised control trials, are not appropriate for most public health exposures. This is chiefly because randomisation to many public health exposures of interest, such as smoking, alcohol, or early age at first pregnancy, is often either impractical or unethical. Well designed observational studies are often the best source of evidence on public health issues.

The available evidence had several limitations. Well known associations (eg, between alcohol and cirrhosis) were often observed in older research, when standards of documentation of methods were different. Also, very heavy drinkers are typically under‐represented in cohorts. Reverse causation can complicate interpretation of findings; for example, people who have become sick or have developed a problem with alcohol may reduce their alcohol consumption. There is also limited evidence on the impacts of lower levels of consumption among youth and on the effects of alcohol on children whose mothers drink alcohol while breastfeeding.

#### Public call for evidence

The NHMRC made an early public call for evidence submissions on health risks and benefits of alcohol consumption to complement the systematic reviews, mathematical modelling, and other evidence considered. This call helped identify relevant studies and gaps in the evidence, as well as areas of concern for stakeholders. Clear inclusion and exclusion criteria ensured that only studies of reasonable quality were accepted.

As the additional literature was collected outside the systematic review process, it could not be evaluated using the GRADE approach applied to the systematic reviews. Instead, the evidence from the public call was considered along with evidence from the 2009 guidelines, systematic reviews, modelling and other evidence and contributed to the narrative text for the guidelines.

#### Additional articles

Additional articles of particular significance were also considered; for example, if they were published after the last systematic review. A transparent process was implemented for choosing and assessing the quality of additional articles. These articles included Mendelian randomisation studies, recent major cohort studies, experimental studies, and imaging studies on brain development among youth. Such articles could not be incorporated as core inputs into the guideline development, but they could be used in explanation and as an influencer when decisions were not clearly based on other sources of evidence.

### Mathematical modelling

The mathematical model developed by the School of Health and Related Research at the University of Sheffield^18^ was adapted for Australian alcohol consumption and health data. The model derives the absolute risk of dying from disease or injury related to alcohol for different patterns and levels of drinking. Australian data on drinking patterns and alcohol‐related morbidity and mortality were used as inputs. The consumption threshold, which corresponds to a one in 100 risk of dying from an alcohol‐related cause, was examined.

The committee used the absolute risk of dying from an alcohol‐related cause rather than the relative risk (ie, rather than how many times greater or lower the risk of dying is at different levels of alcohol consumption).

Because current abstainers can differ from drinkers in a range of ways that are likely to bias results, they were not used as a comparison group. Current non‐drinkers, for example, can include past drinkers who stopped drinking as a result of an illness.

### Principles for considering the body of evidence

The Committee agreed the following *a priori* regarding the modelling evidence on alcohol‐related mortality:

• To consider the typical pattern of drinking in Australia when setting guidelines for healthy (non‐pregnant/non‐breastfeeding) adults. Accordingly, the drinking frequency of 3 days per week was used.^1^
• To round down rather than up when deciding on limits, given the growing uncertainty over the cardioprotective effects of alcohol, the ongoing emergence of research showing that the risk of common cancers increases from low levels of consumption, and the need for caution (eg, a limit of 12.5 from the modelling would be considered a limit of 12).
• To provide a guideline that is easy to implement. There was little difference between the lifetime risk of alcohol‐related death in males compared with females at lower levels of consumption (between zero and ten standard drinks per week). Among individuals who drink 3 days per week, the risk of dying from an alcohol‐related cause becomes one in 100 at just over ten standard drinks per week for both men and women. Therefore, there was no compelling reason to change from the approach of the 2009 guidelines, which had the same limit for men and women in the general population.

The committee agreed to use the precautionary principle in relation to pregnancy and breastfeeding, and for those under the age of 18 years, because of evidence of potential major harms to the developing brain from alcohol.

To develop the guidelines, the committee also applied the GRADE Evidence to Decision frameworks.^13^ As part of this process, the committee considered the quality and certainty of the evidence, the values and preferences of the target population, the impact of the recommendation on health equity, the resource implications, and the feasibility and acceptability of the recommendation and other considerations.^1^

### Public consultation and independent expert review

Public consultation is a core feature of the NHMRC’s guideline development, contributing to accountability of the agency and independence of the advice. The draft guidelines were released for a 10‐week public consultation in December 2019. The process was conducted in accordance with Section 13 of the *National Health and Medical Research Council Act 1992*. The online publication of the draft guidelines was accompanied by a media release and the consultation was advertised through the NHMRC website, the NHMRC newsletter, and social media. Correspondence was also sent to a large number of stakeholders inviting them to provide input. In total, 49 public consultation submissions were received. All were considered. No errors of fact or significant omissions of evidence were identified, but phrasing of the guidelines and accompanying resources were clarified at several points.

After revisions in response to public consultation, the guidelines underwent review by four independent experts. The modelling was also reviewed independently by a team of six experts. After feedback from the expert review, the guidelines were further refined.

The final guidelines were released in December 2020 following endorsement by the NHMRC Council.^1^

## Recommendations

After taking into consideration all the sources of evidence, public feedback and expert review, the Committee devised the following guidelines to reduce the risk from alcohol consumption.

### Guideline 1: adults

To reduce the risk of harm from alcohol‐related disease or injury, healthy men and women should drink no more than ten standard drinks a week and no more than four standard drinks on any one day.

The less you drink, the lower your risk of harm from alcohol.

### Guideline 2: children and people under 18 years of age

To reduce the risk of injury and other harms to health, children and people under 18 years of age should not drink alcohol.

### Guideline 3: women who are pregnant or breastfeeding

To prevent harm from alcohol to their unborn child, women who are pregnant or planning a pregnancy should not drink alcohol.

For women who are breastfeeding, not drinking alcohol is safest for their baby.

The guidelines are summarised in Box 3, with examples of standard drinks given in Box 4.

Box 3 **Alcohol guidelines infographic**

**Figure:**
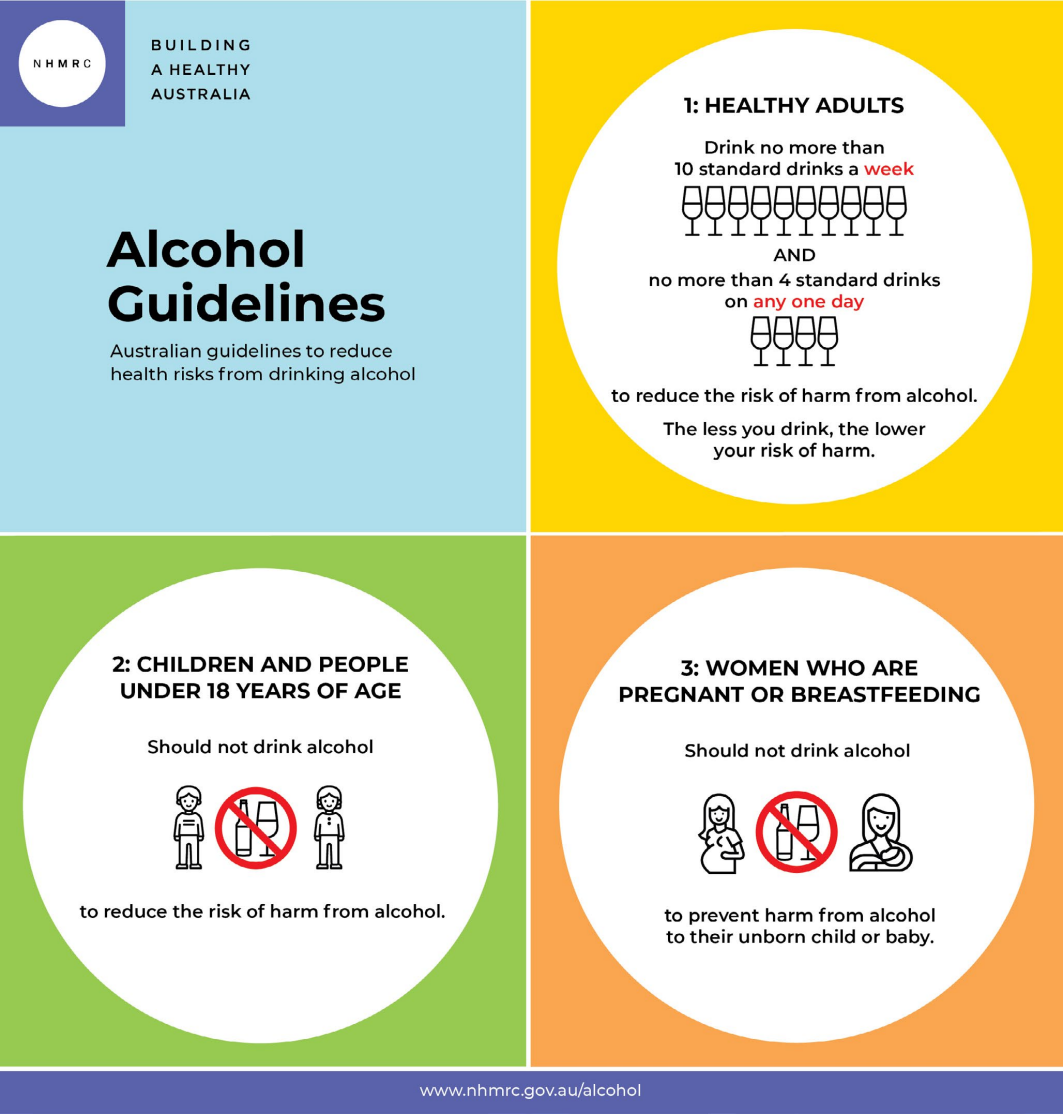
Source: National Health and Medical Research Council.^19^

Box 4 **“What is a standard drink?” infographic**

**Figure:**
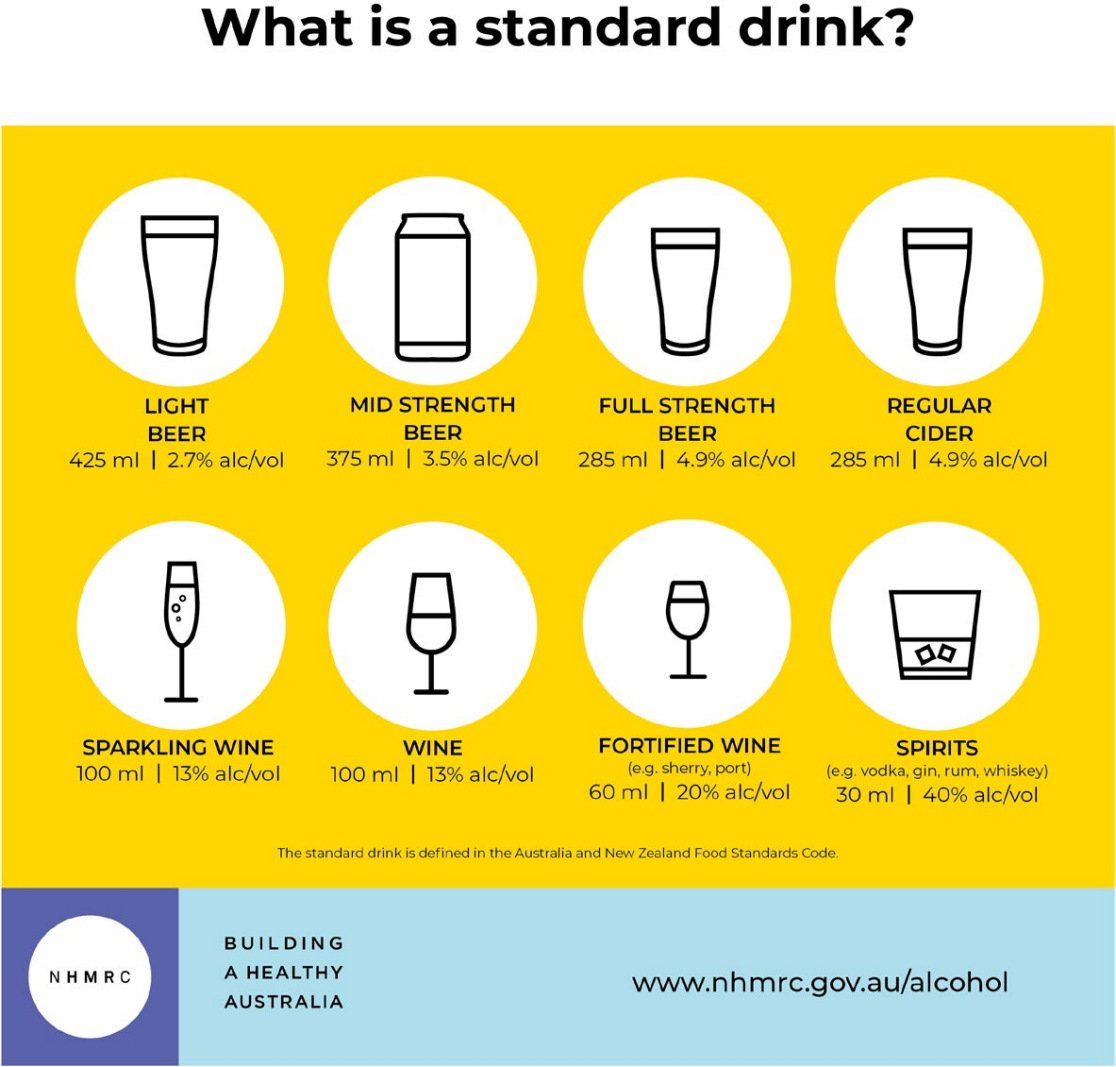
Source: National Health and Medical Research Council.^1^

The key differences from the previous guidelines are that:

• the guidelines for short and long term harm were combined into a single guideline;
• the total weekly recommended consumption limit was effectively changed from 14 standard drinks to ten; and
• the recommended drinking limit for any one day remained the same (at four drinks) but was clarified (it was previously phrased as “per drinking occasion”).

The statement “the less you drink, the lower your risk of harm from alcohol” was added to clarify that any level of drinking can convey some risk of harm. Furthermore, individuals with health conditions are again advised to seek medical advice on drinking. For pregnancy, breastfeeding and people aged under 18 years, the guidance not to drink alcohol was made clearer.

The publication of the guidelines was accompanied by press releases and considerable media coverage. The full guidelines are available online at https://www.nhmrc.gov.au/about‐us/publications/australian‐guidelines‐reduce‐health‐risks‐drinking‐alcohol, and are also published on MAGICApp at https://app.magicapp.org/#/guideline/4630. The Commonwealth Department of Health is responsible for the implementation of the guidelines.

## Competing interests

Until December 2020, Michael Livingston was deputy director of the Centre for Alcohol Policy Research at La Trobe University, which received core funding from the Foundation for Alcohol Research and Education (FARE), a not‐for‐profit organisation working towards an Australia free from alcohol harms.

## Provenance

Not commissioned; externally peer reviewed.
